# Hypercobalaminaemia is associated with hepatic and neoplastic disease in cats: a cross sectional study

**DOI:** 10.1186/s12917-014-0175-x

**Published:** 2014-08-08

**Authors:** Mary R Trehy, Alexander J German, Paolo Silvestrini, Goncalo Serrano, Daniel J Batchelor

**Affiliations:** 1 School of Veterinary Science, University of Liverpool, Leahurst Campus, Chester High Road, Neston CH64 7TE, UK; 2 Department of Obesity and Endocrinology, University of Liverpool, Leahurst Campus, Chester High Road, Neston CH64 7TE, UK

**Keywords:** B12, Vitamin, Feline

## Abstract

**Background:**

When increased serum cobalamin concentrations are encountered clinically they are usually attributed to parenteral supplementation, dietary factors, or otherwise ignored. However, recently, hypercobalaminaemia has been associated with numerous diseases in humans, most notably neoplastic and hepatic disorders. The aim of this retrospective, observational, cross-sectional study was to determine the significance of increased cobalamin in cats.

**Results:**

In total, 237 records were retrieved and 174 cats, of various ages and sexes met the inclusion criteria. A total of 42 cats had increased serum cobalamin concentration, and had not received prior supplementation. Multiple logistic regression analysis revealed that increased serum cobalamin concentration was positively related to pedigree breed (pedigree breeds more likely to have increased cobalamin concentration, odds ratio [OR] 4.24, 95% CI 1.78-10.15, P = 0.001), to having liver disease (OR 9.91, 95% CI 3.54-27.68), and to having a solid neoplasm (OR 8.54, 95% CI 1.10-66.45).

**Conclusions:**

The results of the current study suggest that increased serum cobalamin concentrations should not be ignored in cats with no history of supplementation, and investigation for underlying hepatic or neoplastic disease is warranted.

## Background

Cobalamin is a water-soluble vitamin with important intermediary metabolic functions. The importance of hypocobalaminaemia has long been recognised: deficiency can have neurological, haematological, cardiovascular and catabolic manifestations in man and encephalopathy, myelopathy, anaemia, anorexia, cold-intolerance and failure to thrive have been reported in cats [1],[2]. Hypocobalaminaemia has been reported in cats with gastrointestinal disease [3],[4], exocrine pancreatic insufficiency [5] and hyperthyroidism [6]. Decreased serum cobalamin can help to localise gastrointestinal disease [7] and is a negative prognostic indicator for alimentary lymphoma in cats [8].

Conversely, increased circulating cobalamin concentrations are generally attributed either to increased dietary intake or to previous supplementation and, as a consequence, are usually ignored. However, in the absence of supplementation, hypercobalaminaemia reflects altered metabolism [9]. In humans, hypercobalaminaemia is associated with myeloproliferative disorders [10]-[12] and with a wide range of solid neoplasms including liver, mammary, prostatic, pulmonary, gastric and pancreatic tumours [9],[13]. It has, therefore, been suggested to be a non-specific marker for cancer [13]. Associations have also been made with various non-neoplastic liver diseases [9],[14],[15] and with kidney disease in some studies [16],[17]. To date, its significance has not been investigated in veterinary species. Therefore, the aim of this study was to determine whether disease associations exist for hypercobalaminaemia in cats.

## Methods

### Study design

This was a retrospective, observational, cross-sectional study and has been reported according to the guidelines of the Strengthening the Reporting of Observational Studies in Epidemiology (STROBE) statement [18], see additional information (STROBE Checklist) for further details. The study was performed in adherence to the University of Liverpool Animal Ethics Guidelines.

### Case recruitment and method of case identification

Case records of cats referred to the University of Liverpool Small Animal Teaching Hospital between January 2006 and April 2013 were reviewed. To be eligible for inclusion, cats had to have had serum cobalamin concentration measured at the time they were first referred for investigation, and have a complete medical record available for review.

The hospital clinical records database was first searched using the terms “cobalamin” and “B12”. The case records of each cat identified by these searches were then examined to identify those cases which had had cobalamin measured by solid-phase, competitive, chemiluminescent enzyme immunoassay (Immulite 2000^a^). The reference interval used was 270–1200 ng/L. During the course of the study, the upper limit, to which samples were diluted to obtain an absolute concentration (when cobalamin concentration exceeded the reference interval), changed from 1200, to 1500, then 2000. However, the upper limit of the reference interval itself did not change (i.e. remained at 1200 ng/L throughout).

Information was recorded regarding signalment (e.g. age, sex, neuter status, and breed), date of admission, presenting signs, history of oral or parenteral cobalamin supplementation, clinicopathological results (e.g. serum cobalamin assay, folate, presence of leucocytosis, serum ALT), diagnostic investigations performed, and final diagnosis. Data regarding outcome and current diet were recorded when available.

### Data handling and statistical analysis

Data were not normally distributed and are, therefore, expressed as median (range). Cobalamin concentrations in excess of the upper limit reported at the time of sampling were recorded as 1201, 1501 and 2001, respectively, for statistical analyses. Statistical analysis was performed using computer software^b^, with the level of significance set at *P* < 0.05 for two-sided analyses, and no correction was made for missing data.

Presenting signs and results of diagnostic investigations were compared between groups using the Kruskal Wallis test. The relationship between serum cobalamin and ALT and between serum cobalamin and folate was assessed using the Spearman’s rank correlation coefficient. The proportion of definitive diagnoses made was compared amongst groups with Fisher’s exact test. The effect of signalment (age, sex, breed group) and disease type were evaluated with simple and multiple logistic regression analysis. Initially, simple logistic regression was used, whereby each factor was tested individually. Multiple logistic regression models were then constructed, which initially included variables identified as *P* < 0.30 on univariable analysis. The initial model was subsequently refined, over multiple rounds, by backwards-stepwise elimination of the least significant variable at each round, with variables retained in the final model if they were significant (*P* < 0.05), or where removal lead to a notable reduction (i.e. ≥30%) in the overall fit of the model.

## Results

### Study population

**Figure 1:**
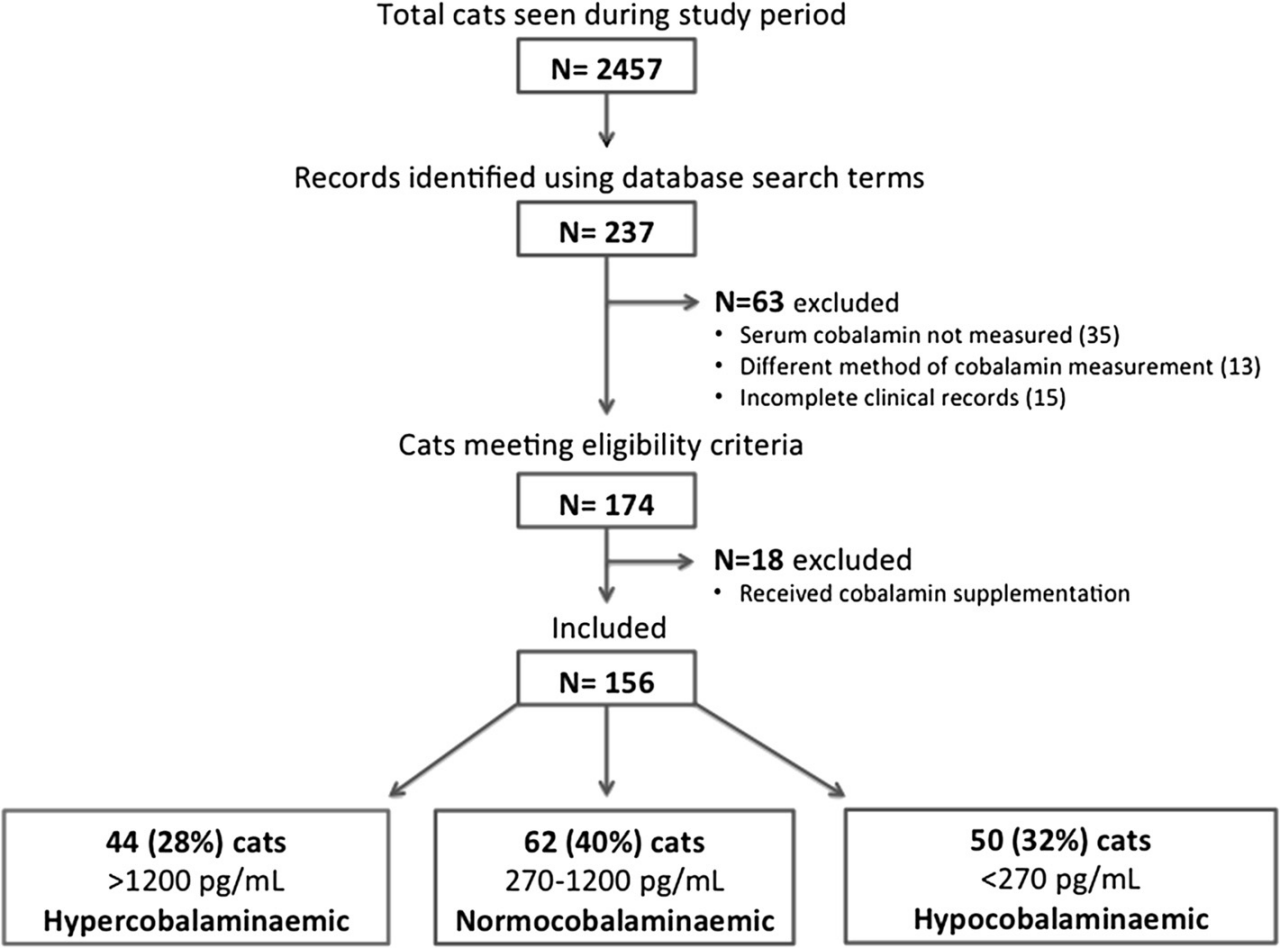
Flow chart of cats included in the study.

During the study period, a total of 2457 cats attended the Small Animal Teaching Hospital. Of these, 237 records were identified after the database search, and 174 cases met the eligibility criteria (Figure 1). Reasons for exclusion were lack of a result for serum cobalamin (35 cats), cobalamin measured using a different method (13 cats), or incomplete clinical records (15 cats). A record of prior cobalamin supplementation (within the three months before presentation) was identified in 18 cases (16 with serum cobalamin greater than the reference interval, 1 with cobalamin within the reference interval and 1 with cobalamin below the reference interval). Among the remaining 156 cats with cobalamin measurement, complete medical records available, and no history of cobalamin supplementation, the serum cobalamin was greater than the reference interval in 44 (28%; hypercobalaminaemia group), within the reference interval in 62 (40%; normocobalaminaemia group), and below the reference interval in 50 (32%; hypocobalaminaemia group) cases. In 12 of the hypercobalaminaemic cats, the absolute cobalamin concentration was determined by serial dilution as 1597 ng/L (1507 to 1892). Of the remaining 32 hypercobalaminaemic cats, 6 were reported as >2000 ng/L, 4 as >1500 ng/L and 22 as >1200 ng/L.

### Characteristics of the study cats

#### Signalment

Signalment of the enrolled cats is summarised in Table 1. More cats in the hypercobalaminaemia group were from pedigree breeds (22/44) than normocobalaminaemic (19/62) and hypocobalaminaemic (15/50) cats (*P* = 0.02). The median age did not differ between the groups (*P* = 0.59).

**Table 1:** Signalment of study cats

**Group**	**Age**	**Breed**		**Gender**	
		**Pedigree breeds**	**Domestic shorthair**	**Male (entire)**	**Female (entire)**
Hypercobalaminaemia (44)	9y2m (7 m - 18y 3 m)	22	22	30 (3)	14 (0)
		5 Maine Coon, 4 Bengal ,3 Birman, 2 Burmese,2 Ragdoll, 2 Tonkinese, 1 Persian, 1 Oriental, 1 Siamese, 1 Sphynx			
Normocobalaminaemia (62)	11y5m	19	43	36 (7)	26 (2)
	(2 m - 16y 2 m)	6 British Shorthair, 4 Siamese, 2 Burmese, 2 Selkirk Rex, 1 Asian, 1 Maine Coon, 1 Norwegian Forest Cat, 1 Persian, 1 Ragdoll			
Hypocobalaminaemia (50)	12y1m	16	34	41 (2)	9 (1)
	(3 m - 10y 5 m)	3 Siamese, 2 British Shorthair, 2 Burmese, 2 Maine Coon, 2 Oriental, 2 Ragdoll, 1 Korat, 1 Norwegian Forest Cat, 1 Persian			

Age data expressed as median (range).

#### Clinical signs

Presenting signs reported for cats in all groups included weight loss, vomiting, anorexia, diarrhoea and lethargy and the frequency with which each sign was reported did not differ between groups (Table 2).

**Table 2:** Presenting signs of study cats by group

**Group**	**Number of cats displaying sign**				
	**Vomiting**	**Diarrhoea**	**Lethargy**	**Inappetance**	**Weight Loss**
Hypercobalaminaemia (44)	17	14	10	16	21
Normocobalaminaemia (62)	26	24	8	15	26
Hypocobalaminaemia (50)	24	20	11	8	30
Significance (*P*)*	0.67	0.74	0.30	0.06	0.18

*All comparisons made using the Kruskal Wallis test.

#### Diagnostic investigations

The results of concurrent haematological, serum biochemical and serum folate analyses were available in all cases. There was no difference in the proportion of cats with leucocytosis (*P* = 0.72), increased ALT activity (*P* = 0.18), or decreased serum folate concentration (*P* = 0.69) between groups, and there was no significant association between serum cobalamin and either ALT (R_s_ = −0.06; *P* = 0.53) or serum folate concentration (R_s_ = −0.058; *P* = 0.58). All cats underwent thoracic radiography and abdominal ultrasonography and 22, 28 and 29 cats in the hypercobalaminaemia, normocobalaminaemia and hypocobalaminaemia groups, respectively, had cytological or histopathological evaluation of lesions performed.

#### Diagnosis

A definitive diagnosis was recorded in 33/44 (75%) cats in the hypercobalaminaemia group, 40/62 (65%) cats in the normocobalaminaemia group and 41/50 (82%) cats in the hypocobalaminaemia group. There was no difference in the proportions of cats with a definitive diagnosis amongst groups (*P* = 0.17). The diagnoses are summarised in Table 3. Of the 33 hypercobalaminaemic cats with a definitive diagnosis, 15 were diagnosed with neoplasia, and 8 were diagnosed with non-neoplastic liver disease. Of the 15 hypercobalaminaemic cats with neoplastic disease, liver involvement was confirmed in 5 cases (2 cats with lymphoma, one cat with a metastatic splenic plasma cell tumour, one cat with a pancreatic carcinoma and one with a biliary cystadenoma). Of the 10 normocobalaminaemic and the 14 hypocobalaminaemic cats diagnosed with neoplasia, liver infiltration was identified in 1 cat in each group (both cats diagnosed with lymphoma).

**Table 3:** Definitive diagnoses* recorded for study cats

**Group**	**Neoplasia**	**Hepatobiliary disease**	**Kidney disease**	**Chronic enteropathy**	**Other**
Hypercobalaminaemia	15	8	2	3	5
(n = 33)	Lymphoma (10)^¶^	Neutrophilic cholangitis/	CKD (2)		Triaditis (1), FIP (1)
	Duodenal adenocarcinoma (2)	cholangiohepatitis (5)			Tritrichomonas (2)
	Pancreatic carcinoma (1)	Hepatic lipidosis (2)			Idiopathic hypercalcaemia (1)
	Biliary cystadenoma (1)	PVH (1)			
	Metastatic plasma cell tumour (1)				
Normocobalaminaemia	10	1	4	15	10
(n = 40)	Lymphoma (8)^¶^	Choledocholithiasis (1)			Tritrichomonas (2)
	Duodenal adenocarcinoma (1)				Diabetes mellitus (1)
	Neuroendocrine mass (1)				FOPS (1) FIV (1)
					HES (1), HCM (1)
					Gastric foreign body (1)
					Pancreatitis (1)
					IMPA (1)
Hypocobalaminaemia	14	0	0	13	14
(n = 41)	Lymphoma (14)^¶^				EPI (5)
					Pancreatitis (3)
					Diabetes mellitus (2)
					Acromegaly (1)
					Pemphigus foliaceus (1)
					Oesophageal stricture (1)
					Intussusception (1)

CKD: chronic kidney disease; EPI: exocrine pancreatic insufficiency; FIV: feline immunodeficiency virus; FIP: Feline infectious peritonitis; FOPS: feline oral pain syndrome; HCM: hypertrophic cardiomyopathy; HES: hypereosinophilic syndrome; IMPA: immune-mediated polyarthritis; PVH: Portal vein hypoplasia. *Definitive diagnoses were made in 33/44 (75%) hypercobalaminaemic, 40/62 (65%) normocobalaminaemic, and 41/50 (82%) hypocobalaminaemic cats. ^¶^For hypercobalaminaemic cats, lymphoma types were: alimentary (gastric 3, small intestinal [including ileal], 5), extranodal (nasal, 1), epitheliotropic (1); for normocobalaminaemic cats, lymphoma types were: alimentary (gastric 1, small intestinal [including ileal] 3, colonic 1, ileocaecolic junction mass 1,), multicentric (1), epitheliotropic (1); for hypocobalaminaemic cats, lymphoma diagnoses were: alimentary (gastric 1, small intestinal [including ileal] 10), combined nasal and small intestinal (1), multicentric (1), epitheliotropic (1).

### Regression analysis to determine factors associated with hypercobalaminaemia in cats

On simple logistic regression analysis (Table 4), the main factors associated with hypercobalaminaemia were breed group (i.e. pedigree cats were more likely to be hypercobalaminaemic; odds ratio [OR] 2.43, 95% confidence interval [95-CI] 1.18-5.00, *P* = 0.02), and liver disease (cats with liver disease more likely to be hypercobalaminaemic, OR 6.78, 95-CI 2.70-17.02, *P* < 0.001). In addition to these variables, gastrointestinal disease, renal disease, all neoplasia, solid neoplasm, and neoplasm in the liver were also included in the initial multiple regression analysis. However, after four rounds of backwards elimination, variables remaining in the final model (Table 5) were breed (pedigree more likely to be hypercobalaminaemic, OR 4.24, 95% CI 1.78-10.15, *P* = 0.001), liver disease (cats with liver disease more likely to be hypercobalaminaemic, OR 9.91, 95% CI 3.54-27.68, *P* < 0.001), and solid neoplasia (cats with a solid neoplasm more likely to be hypercobalaminaemic, OR 8.54, 95% CI 1.10-66.45, *P* = 0.040).

**Table 4:** Simple linear regression analysis to determine factors associated with hypercobalaminaemia in the study cats

**Variable**		**Odds ratio**	**95% confidence interval**	***P*** **value**
Age	Per month	1.00	0.99 to 1.01	0.615
Sex	Female	referent	---	---
	Male	0.84	0.40 to 1.76	0.640
Neuter status	Entire	referent	---	---
	Neutered	1.44	0.38 to 5.43	0.592
Breed	Mixed breed	referent	---	---
	Pedigree	2.43	1.18 to 5.00	0.016
Gastrointestinal disease	Absent	referent	---	---
	Present	0.53	0.26 to 1.09	0.086
Liver disease	Absent	referent	---	---
	Present	6.78	2.70 to 17.02	<0.001
Renal disease	Absent	referent	---	---
	Present	1.87	0.62 to 5.62	0.263
All neoplasia	Absent	referent	---	---
	Present	1.65	0.80 to 3.39	0.174
Solid neoplasia	Absent	referent	---	---
	Present	4.16	0.67 to 25.83	0.126
Neoplasm in liver	Absent	referent	---	---
	Present	8.40	0.85 to 83.10	0.069

**Table 5:** Multiple linear regression analysis to determine factors associated with hypercobalaminaemia in the study cats

**Variable**		**Odds ratio**	**95% confidence interval**	***P*** **value**
Breed	Mixed breed	referent	---	---
	Pedigree	4.24	1.78 to 10.15	0.001
Liver disease	Absent	referent	---	---
	Present	9.91	3.54 to 27.68	<0.001
Solid neoplasm	Absent	referent	---	---
	Present	8.54	1.10 to 66.45	0.040

## Discussion

This study is the first to examine the significance of increased serum cobalamin in cats and the findings suggest that hypercobalaminaemic cats were more likely to be diagnosed with either a solid neoplasm or liver disease. Similar patterns are described in the human literature [9],[13]-[15],[19]-[21], and there is also a recent, as of yet, unpublished study evaluating cobalamin status in dogs with liver disease [22]. Therefore, the current assumption that hypercobalaminaemia does not have clinical significance in veterinary species should be questioned, and prospective studies should be considered to confirm these preliminary findings.

A similar association between hypercobalaminaemia and the presence of a solid neoplasm has been recognised in man [23], with the strongest associations for liver tumours and hepatic metastases [14],[15],[17],[24],[25]. Recently, associations with a wider variety of malignancies have been established [9],[13]. Unfortunately, in the current study, there were insufficient cases presenting with a solid neoplasm to enable associations with any particular tumour type. The reason for the hypercobalaminaemia in the cats with a tumour in the current study is not known. In humans, various mechanisms have been proposed, including the direct production of cobalamin binding proteins (transcobalamins) [9], upregulation of folate-centred metabolic pathways and production of cobalamin autoantibodies [13],[26]. In humans, the association between hypercobalaminaemia and neoplasia was first reported for haematopoietic malignancies, particularly chronic myeloid leukaemia [10]-[12]. Increased production of binding proteins (specifically, a transcobalamin derived from granulocyte membranes) is proposed to occur as a result of leucocytosis [21],[27]. No association with either haematogenous neoplasms or leucocytosis was detected in this study. This might be due to differences in human and feline transcobalamins, namely that cats lack transcobalamin I [28], or might reflect the type of haematogenous neoplasms encountered. The vast majority in this study were lymphoma (32/33) and there is conflicting evidence regarding an association with lymphoma in man. Some [17] but not other [11],[15],[16],[21] studies have suggested an association with hypercobalaminaemia. Additionally, as many of the cats with lymphoma had ileal involvement (the site of intestinal cobalamin absorption), this would likely be confounding, even if a true association with lymphoma does exist.

Hypercobalaminaemic cats in this study were also more likely to be diagnosed with liver disease, and similar associations are seen in humans [9],[14],[15]. Possible mechanisms include leakage into the circulation following hepatocellular injury and impaired hepatic uptake of cobalamin. An association with hepatocellular injury is less likely, given that there was no correlation between serum ALT activity and cobalamin concentration. The association between hypercobalaminaemia and hepatobiliary disease in cats is intriguing, since hepatobiliary disease is also associated with hypocobalaminaemia in this species [3],[4],[7],[28]-[30]. This discrepancy might reflect the tendency for concurrent gastrointestinal, pancreatic and hepatobiliary pathology in cats, having counteracting effects on serum cobalamin. Indeed, only 9 of the 17 hypercobalaminaemic cats with confirmed liver disease in this study had a disease confined to the hepatobiliary system. Alternatively, it could reflect differing effects of specific hepatobiliary diseases on cobalamin metabolism. A final observation, with regard to hepatic disease was the fact that one of the cats had portal vein hypoplasia. The reason why hypercobalaminaemia would be associated with a congenital disease is not known. One possibility might be that there are effects on cobalamin metabolism as a result of changes in hepatic function caused by the PVH. However, further work would be required to determine this.

Associations between kidney disease and hypercobalaminaemia have been identified in some human studies [16],[17], and decreased clearance of binding proteins from the circulation has been postulated [9]. Such an association has not been noted in all human studies [21], and there was no significant difference in serum cobalamin between dogs with chronic kidney disease and controls [31]. Although no such association was found in the current study, only six cats with kidney disease were included. Besides associations with disease, cats of pedigree breeds were more likely to be hypercobalaminaemic. In another study, hypocobalaminaemia was more frequently observed in pedigree breeds [28]. The reason for these findings is unknown.

This study has several limitations. First, methylmalonic acid (MMA) was not measured in any cat as part of the diagnostic investigations, and such results might have helped to clarify cellular cobalamin status. Although it might have been possible to measure MMA retrospectively, in cats where stored serum was available, variability in sample collection procedures (e.g. differing times between collection and freezing of samples), time and temperature of storage could have made the results less reliable. To address this limitation, we would recommend that a future study consider measuring both cobalamin and MMA in cats where methods of collection, handling and storage are standardised.

A second limitation was that data were collected retrospectively and, as a consequence, we were reliant upon the history taken at the time of presentation. Thus, the information acquired from the owner was variable, and this might have been incomplete, for instance information regarding prior cobalamin supplementation. A further limitation of the retrospective design was the fact that, despite a relatively large cohort of cats, relatively few were diagnosed with the diseases most strongly associated with hypercobalaminaemia in man. Further, even within disease categories, there was great diversity of the disease conditions; this was most marked for the two disease categories where an association with hypercobalaminaemia was noted, namely hepatobiliary diseases and solid neoplasms. To address these concerns, we would now recommend conducting a prospective study to assess cobalamin status in a more defined cohort of cats, i.e. with more uniform disease status. The results of the current study could be useful to determine an appropriate sample size to ensure that the project is not underpowered [32]. Further, a multicentre study or collaborative effort would be recommended to ensure that a larger enough cohort is recruited.

A final limitation is the issue of reference interval, not least given the wide variation in reference intervals amongst diagnostic laboratories for serum cobalamin in cats, despite widespread use of the same analytical assay. Reasons for such laboratory variability have previously been proposed in both cats and people, with regional variability, dietary factors, age and undiagnosed disease in control populations suggested as possible explanations [30]. The upper limit of the reference interval used in the current study is less than in some previously reported studies [33],[34] but comparable to others [3],[35]. This interval was based upon that used at other laboratories using the same assay, and then further validated by measuring serum cobalamin concentrations in at least 20 healthy cats over 1y age. Unfortunately, given the validation work was undertaken at least 10 years ago, full details are no longer available. For future studies, it would be advisable for all laboratories to review their reference ranges for cobalamin, and ensure that it is appropriate.

## Conclusion

The current study has suggested possible associations between hypercobalaminaemia and the presence of either solid neoplasia or liver disease in cats. This suggests that veterinarians should pay greater attention to increased serum cobalamin concentrations when investigating sick cats and should consider thorough evaluation for neoplasia or hepatic disease where there is no evidence of prior supplementation.

## Endnotes

^a^Immulite 2000 Vitamin B12; Siemens Healthcare Diagnostics Inc., Deerfield, IL.

^b^StatsDirect version 2.7.9, StatsDirect Ltd, Altrincham, UK.

## Abbreviations

MMA: Methylmalonic acid

## Competing interests

AJG’s academic post at the University of Liverpool is financially supported by Royal Canin.

## Authors’ contributions

MT conceived the study, was involved in data acquisition and produced the original draft of the manuscript. AJG performed statistical analyses and revised the manuscript. PS contributed to interpretation of data and revision of the manuscript. GS was involved in acquisition and interpretation of data. DB contributed to study design, interpretation of data and manuscript revision. All authors read and approved the final manuscript.
